# Construction of a nomogram prediction model for the pathological complete response after neoadjuvant chemotherapy in breast cancer: a study based on ultrasound and clinicopathological features

**DOI:** 10.3389/fonc.2025.1459914

**Published:** 2025-03-06

**Authors:** Pingjuan Ni, Yuan Li, Yu Wang, Xiuliang Wei, Wenhui Liu, Mei Wu, Lulu Zhang, Feixue Zhang

**Affiliations:** ^1^ Department of Ultrasound, The Second Hospital, Cheeloo College of Medicine, Shandong University, Jinan, Shandong, China; ^2^ Department of Pathology, the Second Hospital, Cheeloo College of Medicine, Shandong University, Jinan, Shandong, China

**Keywords:** breast cancer, neoadjuvant chemotherapy, pathological complete response, ultrasound, pathology, nomogram

## Abstract

**Objective:**

To explore the application value of ultrasound in evaluating the efficacy of neoadjuvant chemotherapy (NAC) for breast cancer and construct a nomogram prediction model for pathological complete response (pCR) following different cycles of NAC based on ultrasound and clinicopathological features, and further investigate the optimal prediction cycle.

**Methods:**

A total of 249 breast cancer patients who received NAC were recruited. Ultrasound assessment was performed before NAC and after two cycles of NAC (NAC2), four cycles of NAC (NAC4), and six cycles of NAC (NAC6). All patients underwent surgical resection after NAC6 and the samples were sent for histopathological and immunohistochemical examination. Clinical efficacy was determined according to the Response Evaluation Criteria in Solid Tumors (RECIST). Pathological efficacy was determined according to the Miller-Payne evaluation system (MP); grade 5 was classified as pCR group, while Grades 1-4 were classified as the non-pCR group (npCR). The patients were randomly divided into the training set and the validation set at a ratio of 7:3. The ultrasound and clinicopathological features of the training set were compared, and a nomogram prediction model was constructed based on these features. Finally, the ROC curve, calibration curve, and DCA were used for verification.

**Result:**

Among the 249 patients, 71 (28.5%) achieved pCR, whereas the remaining 178 (71.5%) exhibited npCR. The maximum tumor diameter measured by ultrasound after NAC6 was 1.20 (0.70, 2.10) cm, which was significantly positively correlated with the maximum tumor diameter measured by pathology after surgical resection (*r*=0.626, *P*<0.05). In the training set, multivariate logistic regression analysis revealed that tumor size, posterior echo, RECIST evaluation, and PR status were significantly correlated with pCR after NAC2, NAC4, and NAC6 (*P*<0.05). These indicators were incorporated into static and dynamic nomogram models, demonstrating high predictive performance, calibration, and clinical value in both the training and validation sets.

**Conclusion:**

Regardless of the cycle of NAC, patients with a small tumor, no posterior shadow, a valid RECIST, and a negative PR were more likely to achieve pCR. Evaluation after NAC2 can provide early predictive value in clinical practice.

## 1 Introduction

According to the latest global cancer burden data released by the International Agency for Research on Cancer (IARC),female breast cancer was the second leading cause of global cancer incidence in 2022, with an estimated 2.3 million new cases,comprising 11.6% of all cancer cases (1). Breast cancer imposes a heavy burden on families and society; therefore, research on the diagnosis and treatment of breast cancer holds significance. In clinical practice, early breast cancer lesions can be treated by direct surgical resection. However, in breast cancer cases with a large primary lesion or early metastasis, the therapeutic efficacy of direct surgical resection remains limited. Hence, neoadjuvant chemotherapy (NAC) has emerged as a standard treatment method for most breast cancers. NAC refers to systemic chemotherapy, aiming to reduce the size of the primary tumor, prior to surgical resection. NAC has been shown to have many advantages (2–5), including reducing the clinical stage of breast cancer patients, changing inoperable locally advanced breast cancer into operable breast cancer, and increasing the chance of breast-conserving surgery. Furthermore, NAC offers an opportunity to explore the biological effects of anti-cancer drugs *in vivo* and determine drug sensitivity and/or resistance. Hence, NAC is being increasingly adopted in breast cancer treatment and is recognized as a valuable research platform. Relevant studies (6–8) have shown that achieving pathological complete response (pCR) after NAC can significantly prolong the disease-free survival, event-free survival, and overall survival of patients. Nonetheless, NAC is not effective in all breast cancer patients and residual lesions may persist after NAC (9, 10). Therefore, evaluating the efficacy after NAC is crucial to avoid ineffective chemotherapy in patients who are insensitive to the drug regimen, prompting timely adjustments in the treatment regimen.

At present, histopathology is the gold standard for evaluating the efficacy of NAC, but can only be obtained after the completion of NAC and surgery. The invasive nature of the procedure and the potential delay in treatment represent significant clinical challenges. Therefore, an early, non-invasive, and accurate evaluation method is urgently needed to predict the possibility of patients achieving pCR. Guidelines of the Chinese Society of Clinical Oncology (CSCO) (11) suggest that imaging examinations such as ultrasound, X-ray, computed tomography (CT), and magnetic resonance imaging (MRI) can be used to evaluate clinical tumor response before and after NAC treatment for breast cancer. Anderson et al. (12)employed optical mammography to assess an individual’s response to NAC at the midpoint of treatment. Lee et al. (13) used strict MRI criteria to predict pCR after NAC more accurately. In addition, changes in the apparent diffusion coefficient (ADC) of breast tumors on diffusion-weighted MRI can also predict pCR (14). A systematic review and meta-analysis (15) also compared the accuracy of MRI and PET/CT in assessing NAC pathological responses in breast cancer, revealing that MRI had greater predictive sensitivity and PET/CT had greater specificity. Compared with the above imaging methods, ultrasound has the advantages of convenient operation, no radiation exposure, low cost, dynamic repeatability, and no obvious contraindications. Hence, ultrasonography is widely used in the examination of breast diseases. Cui et al. (16) used ultrasound to evaluate the changes in tumor size after two cycles of NAC, and Wang et al. (17) evaluated the changes in tumor size after the completion of the entire NAC process. Both studies found that the changes in tumor size could provide references for predicting the efficacy of NAC. However, there have been few studies on the dynamic changes of tumor size in the same patient during the entire NAC cycles, and it is not yet clear as to which cycle tumor size change can better predict the efficacy of NAC.

A single factor cannot specifically predict pCR. A nomogram is a comprehensive prediction tool that can be used to predict the probability, risk, and prognosis of the disease by combining multiple risk factors in clinical settings (18, 19). This study employed ultrasound to evaluate the changes in tumor size after different cycles of NAC and constructed a prediction model of pCR after different cycles of NAC based on ultrasound and clinicopathological characteristics. The model was evaluated and validated, and the optimal cycle for predicting pCR was discussed.

## 2 Materials and methods

### 2.1 Participants and study design

**Figure 1:**
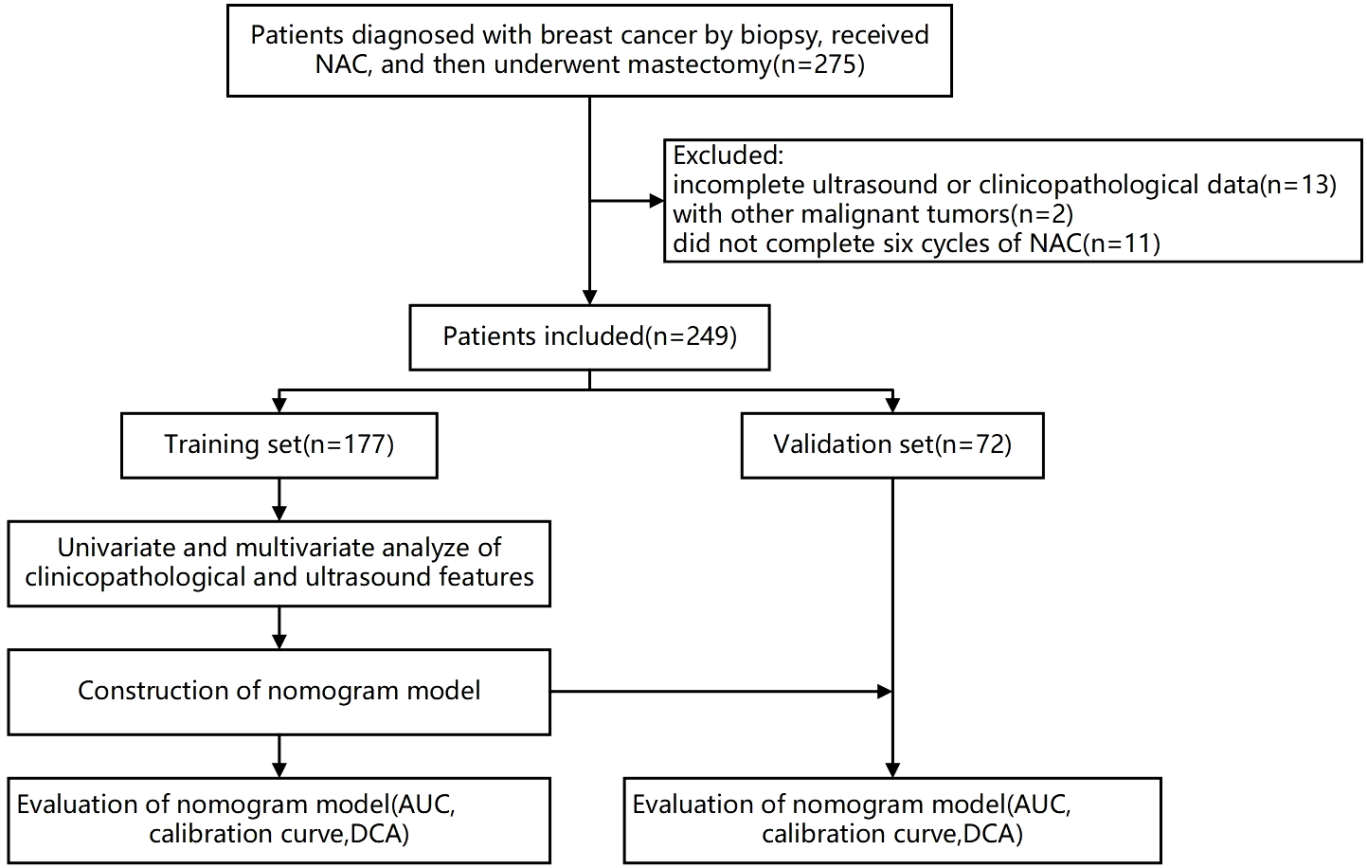
Flowchart of this study.

A total of 249 patients who received NAC in our hospital from January 2020 to July 2023 were recruited. Inclusion criteria: (1) All patients had unilateral primary breast cancer confirmed by biopsy before NAC and underwent a mastectomy after the NAC6 cycle; (2) Both biopsy and excision specimens were subjected to pathological examination and immunohistochemistry (IHC); (3) Ultrasound examinations were performed before NAC and after NAC2, NAC4, and NAC6 cycles, and the data were complete. Exclusion criteria: (1) Incomplete ultrasound or clinicopathological data; (2) Patients with other malignant tumors or inoperable for other reasons; (3) The patient did not complete six cycles of NAC. The 249 patients were randomly divided into a training set and a validation set at a ratio of 7:3. Ultrasound images were collected from ultrasound workstations, and clinicopathological information was obtained from the inpatient electronic medical record system. The time interval was defined as the time from the onset of the first symptoms to the start of treatment. The flowchart of this study is shown in **Figure 1**.

### 2.2 Ultrasonic examination

LOGIQ E9 color ultrasonic diagnostic instrument (GE Healthcare,Wauwatosa, WI) and a linear array probe with a frequency of 6-15MHz were used for ultrasound examination before NAC and after NAC2, NAC4, and NAC6 cycles. The tumor location, size, boundary, aspect ratio, calcification, blood perfusion, and posterior echo were recorded in detail. According to the clinical T stage, the tumor size of the patients was divided into T1 stage (≤2cm), T2 stage (>2cm, ≤5cm), and T3 stage (> 5cm). According to the Adler (20) semi-quantitative blood flow grading method, tumor blood perfusion was classified into grades 0-III. Grade 0: no blood flow; Grade I: minimal blood flow with 1-2 small punctate vessels; Grade II: moderate blood flow with one main vessel and/or several small vessels; Grade III: abundant blood flow with 4 or more vessels visible. According to the Response Evaluation Criteria in Solid Tumors (version 1.1)(RECIST) (21), the reduction rate of the maximum tumor diameter after NAC was calculated. Complete response (CR) was defined as the complete disappearance of the tumor. Partial response (PR) was defined as a reduction of the maximum tumor diameter ≥30%. Progressive disease(PD) was defined as an increase in the maximum tumor diameter of ≥20%. Stable disease (SD) was defined as a decrease in the maximum diameter of the tumor but not reaching PR, or an increase but not reaching PD. CR and PR were considered valid for RECIST assessment, while SD and PD were considered invalid.

### 2.3 Pathological examination

All patients underwent ultrasound-guided puncture biopsy before NAC, and the specimens were examined by histopathology and IHC. Estrogen receptor (ER) and progesterone receptor (PR) positive were defined as ≥1% tumor cell nuclear staining positive; high proliferation of tumor cell proliferation index (Ki67) was defined as ≥20% tumor nuclei positive. Human epidermal growth factor receptor2 (HER2) positive was defined as IHC3+, or gene amplification confirmed by *in situ* hybridization when 2+. All patients underwent a mastectomy after NAC6. The Miller-Payne (MP) grading criteria (22) were used to evaluate the pathological efficacy compared with the pre-NAC puncture specimens. Grade 1: the overall tumor cells showed no change before and after chemotherapy; Grade 2: Tumor cells decreased slightly after chemotherapy, with a reduction rate < 30%; Grade 3: The reduction rate of tumor cells after chemotherapy lied between 30% and 90%; Grade 4: Tumor cells decreased significantly after chemotherapy, with only small clusters or scattered single cells remaining, with a reduction rate > 90%; Grade 5: No residual tumor cells or only intraductal carcinoma. MP5 is defined as pCR and MP1-4 is defined as non-pCR (npCR).

### 2.4 Statistical analysis

SPSS 20.0 statistical software was used for data analysis. Continuous variables conforming to a normal distribution were expressed as mean ± standard deviation and were analyzed using the Student’s t-test. Continuous variables not conforming to a normal distribution were represented by the median (P25, P75) and were tested by the Mann-Whitney U test. Categorical variables were expressed as numbers and percentages and analyzed by the χ2 test or Fisher’s exact test. Furthermore, logistic regression was performed for multivariate analysis, and Spearman correlation analysis was conducted. R software (version 4.2.3) was used to construct a nomogram prediction model, and the online dynamic nomogram was built with Shiny. Finally, the ROC curve, calibration curve, and DCA were used to evaluate the model. *P*<0.05 was considered statistically significant.

This study was approved by the Ethics Review Committee of the Second Hospital of Shandong University (KYLL-2023LW042). Informed consent was obtained from all patients. All experiments were conducted in accordance with the Declaration of Helsinki and relevant guidelines.

## 3 Results

### 3.1 Clinicopathological and ultrasound features of pre-NAC patients

A total of 249 breast cancer patients receiving NAC met the inclusion criteria and were all women. No statistically significant difference in the baseline characteristics was observed between the training set and the validation set (*P*>0.05), as shown in **Table 1**. The analysis revealed that the mean age of the patients at the time of diagnosis was 49.30 ± 9.90 years old, and the age group of 40-60 years old was the most common, with a total of 171 cases (68.7%). Overall, 60.2% of patients received treatment within 3 months of symptom onset. The majority of tumors were at the T2 stage, with 166 cases (66.7%). A total of 160 cases (64.3%) exhibited unclear boundary, 219 cases (88.0%) had an aspect ratio ≤1, 156 cases (62.7%) had calcification, 184 cases (73.9%) demonstrated grade II-III blood flow, and 198 cases (79.5%) had no change for posterior echo.

**Table 1:** Baseline table.

Variables	Total	Training set	Validation set	*p*
	(n=249)	(n=177)	(n=72)	
Age at diagnosis				0.685
≤40 years	46 (18.5%)	35 (19.8%)	11 (15.3%)	
40-60 years	171 (68.7%)	119 (67.2%)	52 (72.2%)	
>60 years	32 (12.9%)	23 (13.0%)	9 (12.5%)	
Menopausal status				0.428
Premenopausal	136 (54.6%)	100 (56.5%)	36 (50.0%)	
Postmenopausal	113 (45.4%)	77 (43.5%)	36 (50.0%)	
Body mass index				0.544
<24	99 (39.8%)	73 (41.2%)	26 (36.1%)	
≥24	150 (60.2%)	104 (58.8%)	46 (63.9%)	
Time interval				0.803
>3 months	99 (39.8%)	69 (39.0%)	30 (41.7%)	
≤3 months	150 (60.2%)	108 (61.0%)	42 (58.3%)	
Tumor location				0.168
Right	126 (50.6%)	95 (53.7%)	31 (43.1%)	
Left	123 (49.4%)	82 (46.3%)	41 (56.9%)	
Tumor size				0.995
≤2cm	56 (22.5%)	40 (22.6%)	16 (22.2%)	
>2cm,≤5cm	166 (66.7%)	118 (66.7%)	48 (66.7%)	
>5cm	27 (10.8%)	19 (10.7%)	8 (11.1%)	
Boundary				0.945
Distinct	89 (35.7%)	64 (36.2%)	25 (34.7%)	
Indistinct	160 (64.3%)	113 (63.8%)	47 (65.3%)	
Aspect ratio				1.000
≤1	219 (88.0%)	156 (88.1%)	63 (87.5%)	
>1	30 (12.0%)	21 (11.9%)	9 (12.5%)	
Calcification				0.183
Absent	93 (37.4%)	61 (34.5%)	32 (44.4%)	
Present	156 (62.7%)	116 (65.5%)	40 (55.6%)	
Alder degree				0.680
0-I	65 (26.1%)	48 (27.1%)	17 (23.6%)	
II- III	184 (73.9%)	129 (72.9%)	55 (76.4%)	
Posterior echo				0.794
Unchanged	198 (79.5%)	142 (80.2%)	56 (77.8%)	
Shadow	51 (20.5%)	35 (19.8%)	16 (22.2%)	
ER status				1.000
Negative	99 (39.8%)	70 (39.6%)	29 (40.3%)	
Positive	150 (60.2%)	107 (60.5%)	43 (59.7%)	
PR status				0.783
Negative	81 (32.5%)	59 (33.3%)	22 (30.6%)	
Positive	168 (67.5%)	118 (66.7%)	50 (69.4%)	
HER2 status				0.412
Negative	123 (49.4%)	84 (47.5%)	39 (54.2%)	
Positive	126 (50.6%)	93 (52.5%)	33 (45.8%)	
Ki67 status				0.935
≤20%	44 (17.7%)	32 (18.1%)	12 (16.7%)	
>20%	205 (82.3%)	145 (81.9%)	60 (83.3%)	
Change of blood perfusion (N2)				0.069
Stable	129 (51.8%)	88 (49.7%)	41 (56.9%)	
Less	103 (41.4%)	80 (45.2%)	23 (31.9%)	
More	17 (6.8%)	9 (5.1%)	8 (11.1%)	
RECIST (N2)				0.939
Invalid	141 (56.6%)	101 (57.1%)	40 (55.6%)	
Valid	108 (43.4%)	76 (42.9%)	32 (44.4%)	
Change of blood perfusion (N4)				0.065
Stable	77 (30.9%)	52 (29.4%)	25 (34.7%)	
Less	166 (66.7%)	123 (69.5%)	43 (59.7%)	
More	6 (2.4%)	2 (1.1%)	4 (5.6%)	
RECIST (N4)				0.593
Invalid	77 (30.9%)	57 (32.2%)	20 (27.8%)	
Valid	172 (69.1%)	120 (67.8%)	52 (72.2%)	
Change of blood perfusion (N6)				0.768
Stable	70 (28.1%)	52 (29.4%)	18 (25.0%)	
Less	151 (60.6%)	105 (59.3%)	46 (63.9%)	
More	28 (11.2%)	20 (11.3%)	8 (11.1%)	
RECIST (N6)				0.251
Invalid	55 (22.1%)	43 (24.3%)	12 (16.7%)	
Valid	194 (77.9%)	134 (75.7%)	60 (83.3%)	

### 3.2 Ultrasound evaluation of tumors before and after NAC

Ultrasound before NAC revealed that the maximum diameter of the tumor was 2.80 (2.1, 3.90) cm, and Adler blood flow was mostly grade II-III, with 184 cases (73.9%). After NAC6, ultrasound showed that the maximum diameter of the tumor was reduced to 1.20 (0.70, 2.10) cm, and Adler blood flow decreased, with the majority of cases (170 cases, 68.3%) being grade 0-I. After NAC6, 194 cases (77.9%) had valid RECIST, including 30 cases of CR (12.0%) and 164 cases (65.9%) of PR; invalid RECIST was found in 55 cases (22.1%), including 47 cases (18.9%) of SD and 8 cases (3.2%) of PD.

**Figure 2:**
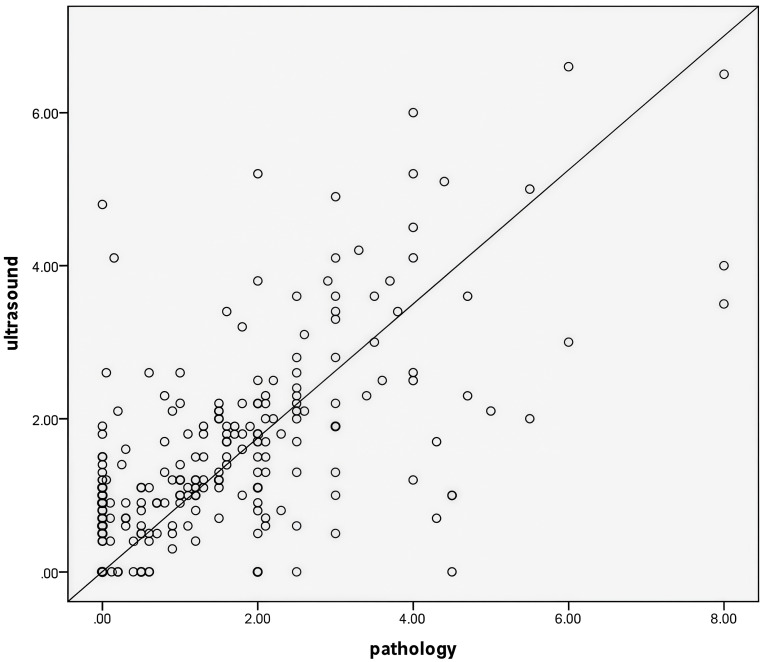
The maximum tumor diameter measured by ultrasound after NAC6 was positively correlated with the maximum tumor diameter measured by pathology(*r*=0.626,*P*<0.05).

The maximum diameter measured by pathology after surgical resection was 1.20 (0.10,2.15) cm, which was positively correlated with the maximum diameter measured by ultrasound after NAC6 (*r*=*0.626*, *P*<0.05) (**Figure 2**).

### 3.3 Correlation between clinicopathological and ultrasonic features of patients and pCR

All patients underwent a mastectomy after NAC6. Pathology showed that 71 cases (28.5%) had MP5 grade and reached pCR, and 178 cases (71.5%) had MP1-4 grade and did not reach pCR. The results of the univariate analysis of the training set showed that tumor size, boundary, posterior echo, blood perfusion changes, RECIST evaluation, and ER, PR, and HER2 expression status were significantly correlated with PCR (*P*<0.05) (**Table 2**). These indicators were incorporated into the multivariate logistic regression of NAC2, NAC4, and NAC6, which showed that tumor size, posterior echo, RECIST evaluation, and PR status were all independent predictors of pCR, as displayed in **Table 3**.

**Table 2:** Univariate analysis of clinicopathological and ultrasonic features in training set.

Variables	*pCR (n=50)*	*npCR (n=127)*	χ^2^	*P*
Age at diagnosis			3.027	0.220
≤40 years	9 (25.7%)	26 (74.3%)		
40-60 years	31 (26.1%)	88 (73.9%)		
>60 years	10 (43.5%)	13 (56.5%)		
Menopausal status			2.047	0.152
Premenopausal	24 (24.0%)	76 (76.0%)		
Postmenopausal	26 (33.8%)	51 (66.2%)		
Body mass index			0.044	0.833
<24	20 (27.4%)	53 (72.6%)		
≥24	30 (28.8%)	74 (71.2%)		
Time interval			3.534	0.060
>3 months	14 (20.3%)	55 (79.7%)		
≤3 months	36 (33.3%)	72 (66.7%)		
Tumor location			1.122	0.289
Right	30 (31.6%)	65 (68.4%)		
Left	20 (24.4%)	62 (75.6%)		
Tumor size			20.753	0.000
≤2cm	22 (55.0%)	18 (45.0%)		
>2cm,≤5cm	27 (22.9%)	91 (77.1%)		
>5cm	1 (5.3%)	18 (94.7%)		
Boundary			5.784	0.016
Distinct	25 (39.1%)	39 (60.9%)		
Indistinct	25 (22.1%)	88 (77.9%)		
Aspect ratio			1.140	0.286
≤1	42 (26.9%)	114 (73.1%)		
>1	8 (38.1%)	13 (61.9%)		
Calcification			0.187	0.665
Absent	16 (26.2%)	45 (73.8%)		
Present	34 (29.3%)	82 (70.7%)		
Alder degree			0.840	0.359
0-I	16 (33.3%)	32 (66.7%)		
II- III	34 (26.4%)	95 (73.6%)		
Posterior echo			8.334	0.004
Unchanged	47 (33.1%)	95 (66.9%)		
Shadow	3 (8.6%)	32 (91.4%)		
ER status			6.088	0.014
Negative	27 (38.6%)	43 (61.4%)		
Positive	23 (21.5%)	84 (78.5%)		
PR status			25.770	0.000
Negative	31 (52.5%)	28 (47.5%)		
Positive	19 (16.1%)	99 (83.9%)		
HER2 status			8.517	0.004
Negative	15 (17.9%)	69 (82.1%)		
Positive	35 (37.6%)	58 (62.4%)		
Ki67 status			0.783	0.376
≤20%	7 (21.9%)	25 (78.1%)		
>20%	43 (29.7%)	102 (70.3%)		
Change of blood perfusion (N2)			10.217	0.006
Stable	17 (19.3%)	71 (80.7%)		
Less	32 (40.0%)	48 (60.0%)		
More	1 (11.1%)	8 (88.9%)		
RECIST (N2)			10.335	0.001
Invalid	19 (18.8%)	82 (81.2%)		
Valid	31 (40.8%)	45 (59.2%)		
Change of blood perfusion (N4)			11.655	0.002
Stable	6 (11.5%)	46 (88.5%)		
Less	44 (35.8%)	79 (64.2%)		
More	0 (0.0%)	2 (100.0%)		
RECIST (N4)			13.028	0.000
Invalid	6 (10.5%)	51 (89.5%)		
Valid	44 (36.7%)	76 (63.3%)		
Change of blood perfusion (N6)			17.604	0.000
Stable	6 (11.5%)	46 (88.5%)		
Less	42 (40.0%)	63 (60.0%)		
More	2 (10.0%)	18 (90.0%)		
RECIST (N6)			10.059	0.002
Invalid	4 (9.3%)	39 (90.7%)		
Valid	46 (34.3%)	88 (65.7%)		

**Table 3:** Multivariate logistic regression analysis in the training set.

Variables	NAC2		NAC4		NAC6	
	OR (95%CI)	*p*	OR (95%CI)	*p*	OR (95%CI)	*p*
Tumor size						
≤2cm	Reference		Reference		Reference	
>2cm,≤5cm	0.227 (0.084,0.617)	0.004	0.219 (0.080,0.597)	0.003	0.195 (0.072,0.530)	0.001
>5cm	0.043 (0.004,0.495)	0.012	0.042 (0.004,0.509)	0.013	0.026 (0.002,0.305)	0.004
Posterior echo						
Unchanged	Reference		Reference		Reference	
Shadow	0.165 (0.041,0.666)	0.011	0.171 (0.039,0.748)	0.019	0.131 (0.030,0.582)	0.008
PR status						
Negative	Reference		Reference		Reference	
Positive	0.181 (0.039,0.853)	0.031	0.137 (0.031,0.612)	0.009	0.166 (0.038,0.734)	0.018
RECIST						
Invalid	Reference		Reference		Reference	
Valid	2.921 (1.210,7.054)	0.017	5.533 (1.679,18.236)	0.005	6.257 (1.586,24.680)	0.009

### 3.4 Construction and verification of the pCR nomogram prediction model

**Figure 3:**
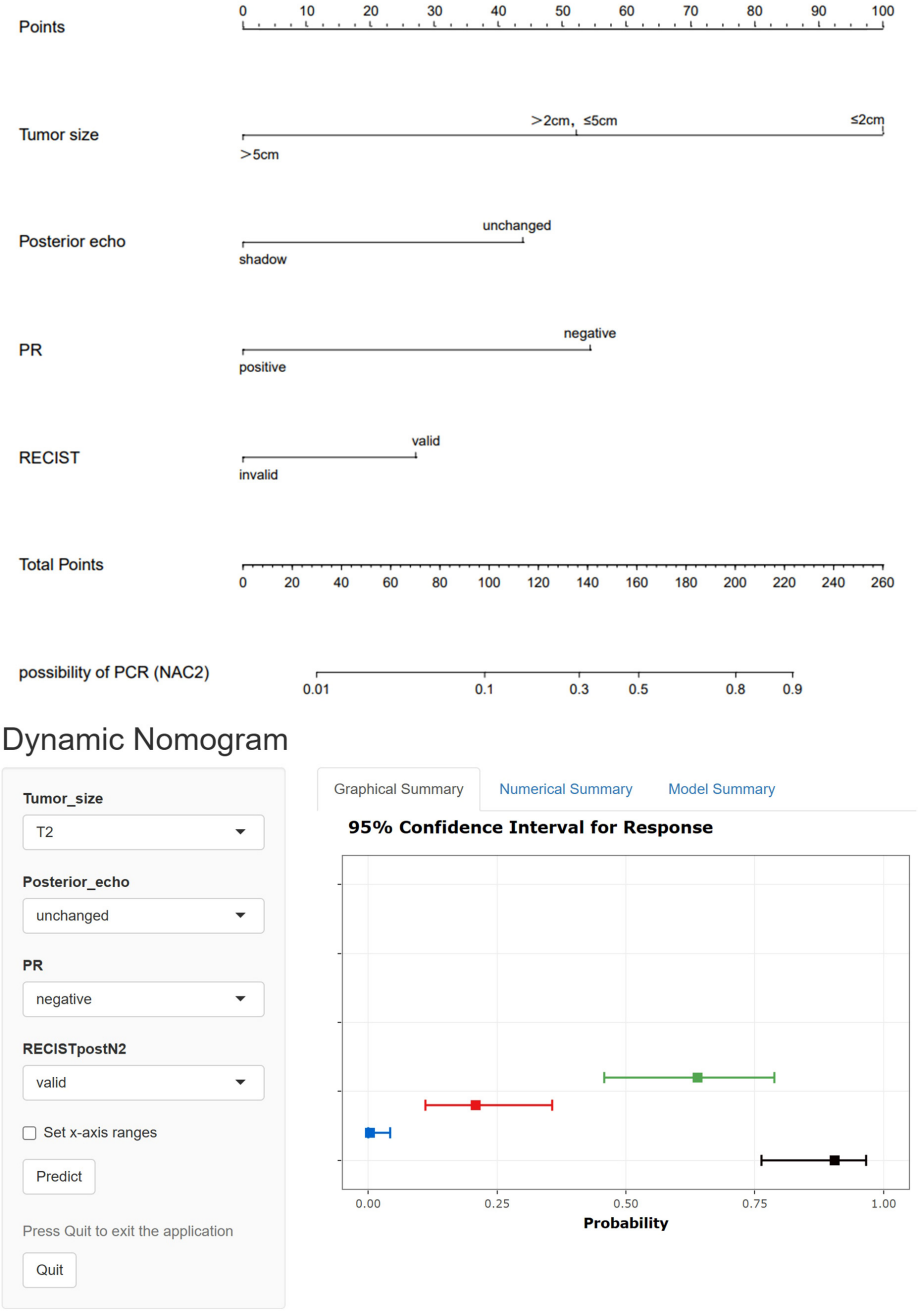
Static and dynamic nomogram model for predicting pCR after NAC2.The dynamic nomogram is available at https://saprediction.shinyapps.io/RECISTPOSTN2/.

**Figure 4:**
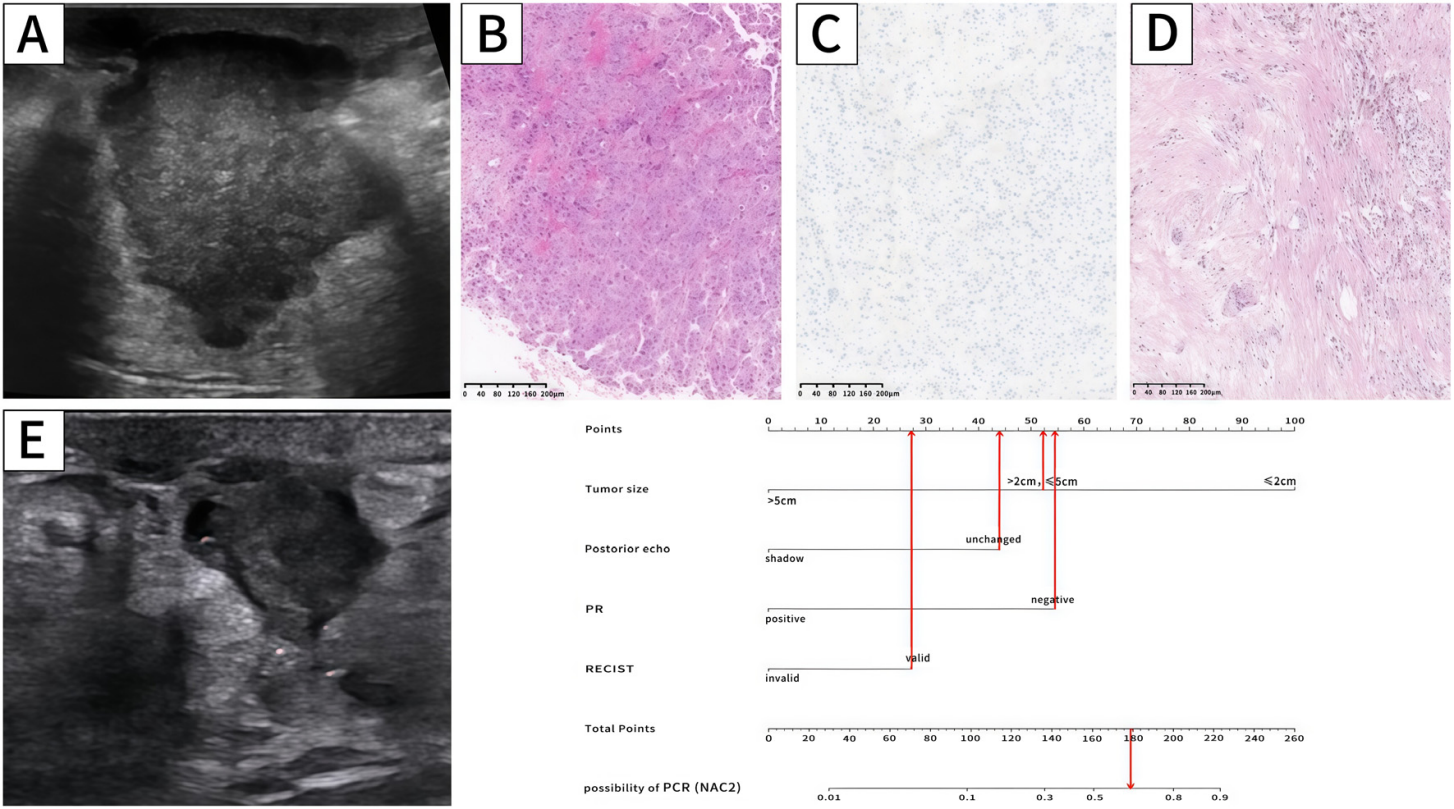
Ultrasound,pathology and nomogram of a breast cancer patient. Ultrasound before NAC **(A)** revealed that the maximum tumor diameter was 3.2cm (52.5 points),and there was no posterior shadow (44 points). Biopsy pathology **(B)** confirmed breast cancer,and IHC **(C)** showed PR (-) (54.5 points).Ultrasound after NAC2 **(E)** showed that the maximum tumor diameter reduced to 1.6cm, and the RECIST was valid (28 points). The total score is 179 (52.5 + 44+54.5+28 = 179 points). The probability of this patient achieving PCR was approximately 0.64. The pathology after mastectomy **(D)** showed MP5 and the patient achieved pCR.

Indicators with statistical differences shown by multivariate logistic regression in the training set were included in static and dynamic nomograms to predict the possibility of pCR after NAC2, NAC4, and NAC6. The static and dynamic nomogram after NAC2 are shown in **Figure 3**, and the dynamic nomogram can be obtained from https://saprediction.shinyapps.io/RECISTPOSTN2/. According to the nomogram, the probability of a tumor achieving PCR after NAC2 can be determined by the sum of the ultrasound and pathological scores (**Figure 4**).

**Figure 5:**
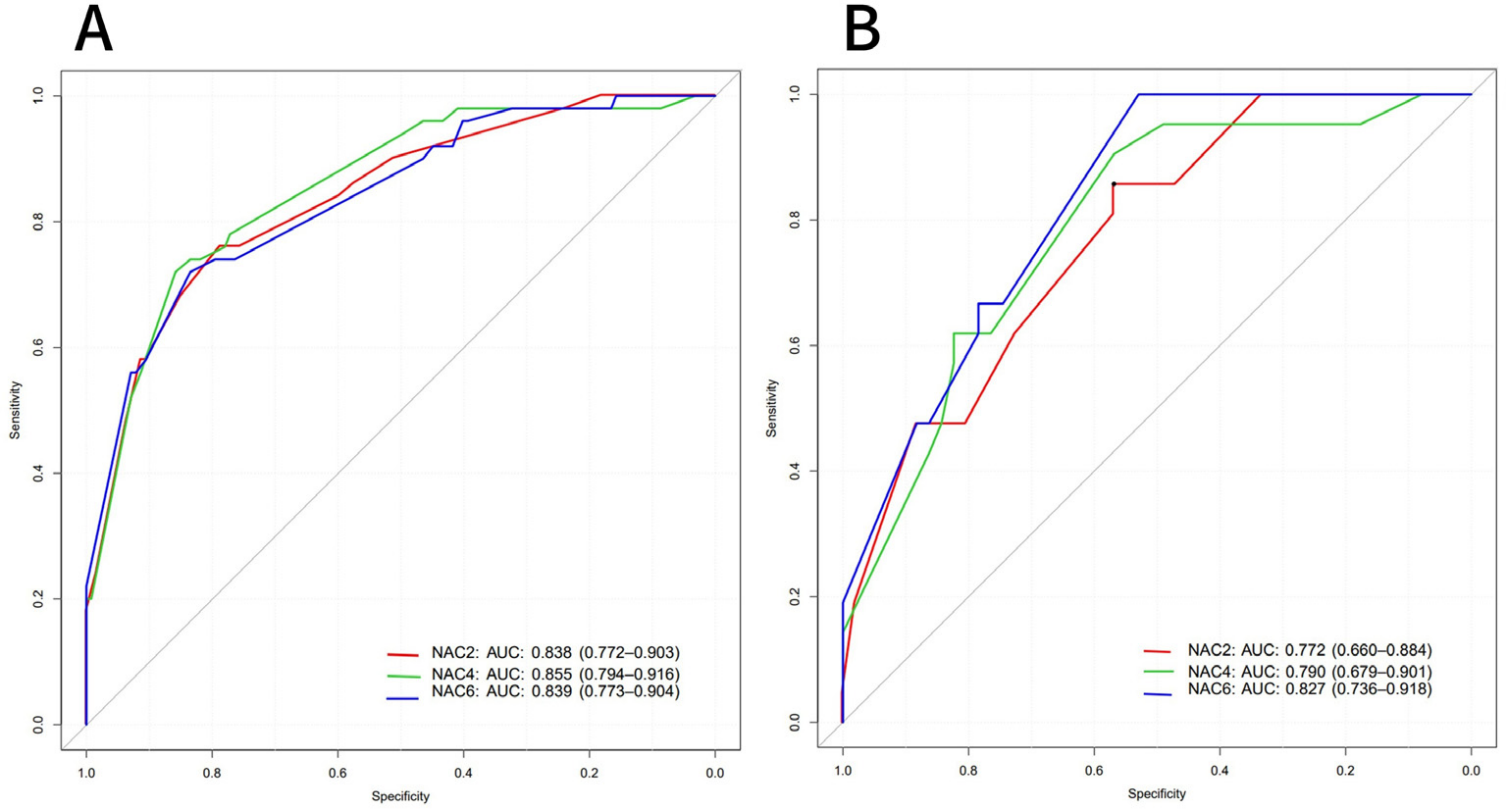
ROC curves of the training set **(A)** and validation set **(B)**.

**Figure 6:**
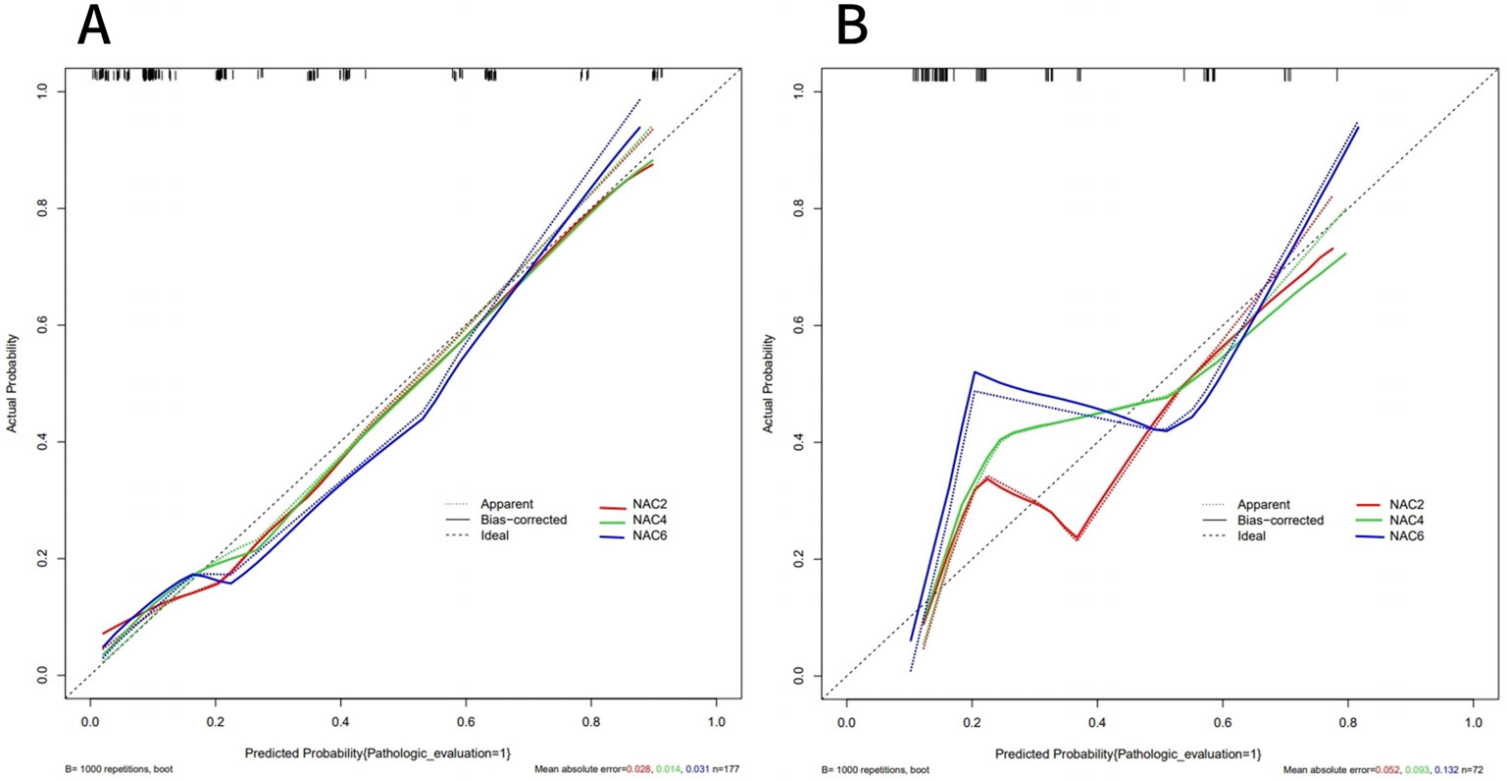
Calibration curves of the training set **(A)** and validation set **(B)**. The calibration curves represents the relationship between the predicted probability of achieving PCR (x-axis) and the actual probability (y-axis), The dashed line on the diagonal represents the predicted probability = the actual probability, and the solid line represents the nomogram calibration curve. The curves of the training and validation set were close to the dashed line, indicating a high degree of calibration.

**Figure 7:**
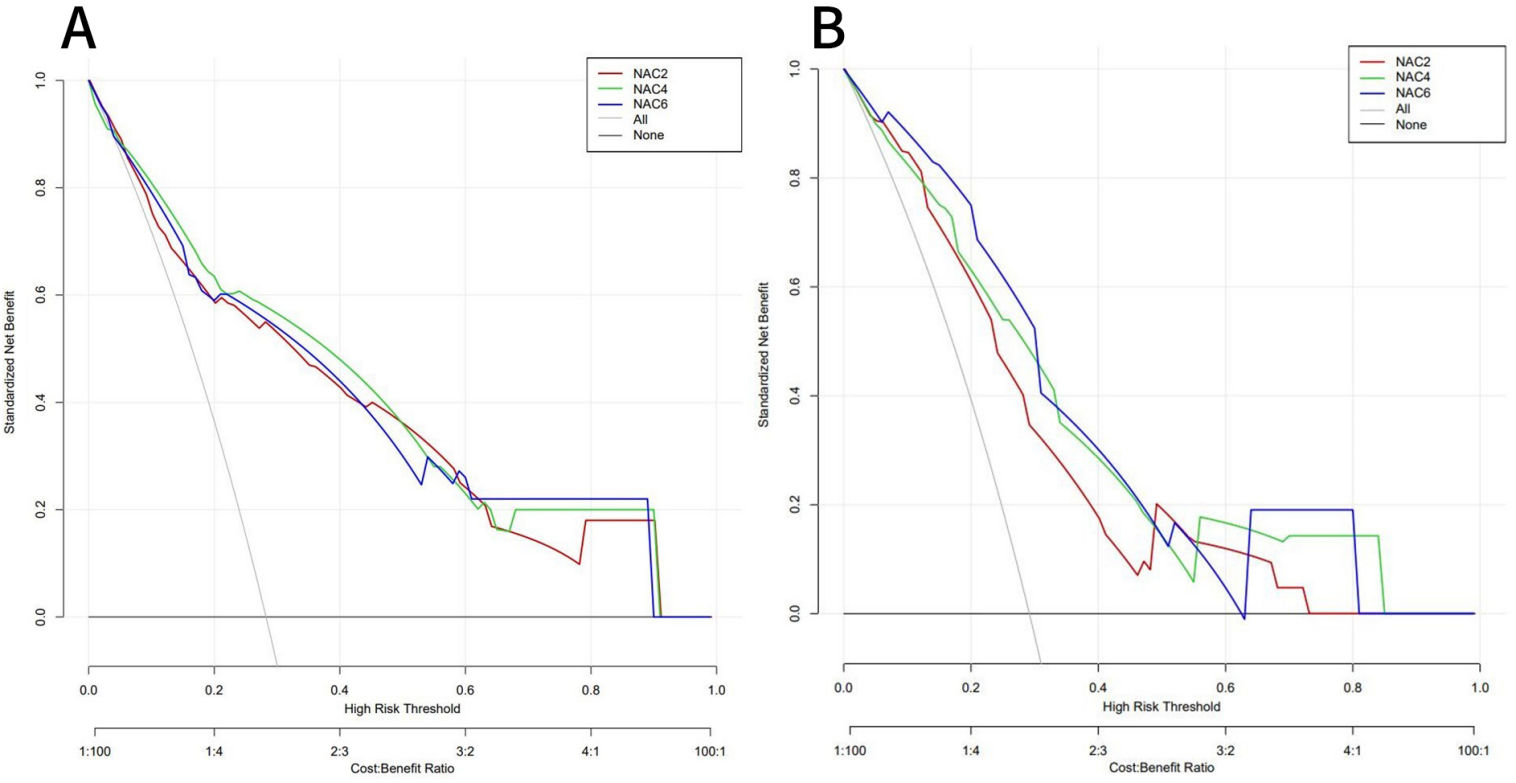
DCA of the training set **(A)** and validation set **(B)**. In both the training set and the validation set,the nomogram exhibited clinical benefits.

ROC curves showed that the AUC of the model in the training set after NAC2, NAC4, and NAC6 were 0.838 (95%CI: 0.772-0.903), 0.855 (95%CI: 0.794-0.916), and 0.839 (95%CI: 0.773-0.904), respectively (**Figure 5A**). The AUCs of the validation set were 0.772 (95%CI: 0.660-0.884), 0.790 (95%CI: 0.679 to 0.901), and 0.827 (95%CI: 0.736 to 0.918), respectively (**Figure 5B**). These results indicated that the model had high predictive performance for pCR after NAC2, NAC4, and NAC6. The calibration curves showed a good agreement between the predicted probability and the actual probability in both the training set (**Figure 6A**) and the validation set (**Figure 6B**). DCA showed a wide range of patient applications and a high clinical benefit in both the training set (**Figure 7A**) and the validation set (**Figure 7B**).

## 4 Discussion

The advantages of NAC have been extensively documented in recent years (2–5). The National Cancer Database (NCDB) also reported that NAC is increasingly being adopted to treat breast cancer (23). Patients who achieved pCR after NAC exhibited a significantly better survival prognosis compared to those with npCR (6–8). However, breast cancer is a heterogeneous disease; not all patients can benefit from NAC, and some patients may develop resistance or even disease progression. Clinical treatment strategies are often developed and revised based on tumor biology and response to systemic therapy. Excessive chemotherapy for NAC-insensitive people not only increases the patient burden but also delays the optimal time for treatment. Therefore, this study aimed to screen out factors that may affect the sensitivity of NAC efficacy. A prediction model was constructed based on pCR after different cycles of NAC. The model allows distinguishing patients with different responses to NAC as early as possible, and then guide clinical personalized and precise treatment.

In this study, MP5 was defined as pCR, and MP1-4 was defined as npCR. The results revealed that the pCR rate was 28.5% and the npCR rate was 71.5%, which was basically consistent with the results of previous relevant studies (16, 24–26).

Our results showed that tumor size, posterior echo, RECIST evaluation, and PR status were all independent influencing factors in predicting pCR after NAC2, NAC4, and NAC6. These four indicators played an important role in the whole process of NAC. Based on these four indicators, pCR prediction models were constructed for the training set after NAC2, NAC4, and NAC6. The results showed that the three models had good prediction performance; the calibration curves also showed good consistency, and the DCA showed a wide application range for patients and a high clinical net benefit. We recommend predicting and evaluating the efficacy of NAC after NAC2, so as to clarify the therapeutic effect as early as possible and provide a theoretical basis for timely adjustment of clinical treatment strategies.

Previous studies (27) compared the tumor size measured by ultrasound in breast cancer patients with the pathological size of resected specimens, and the results showed high consistency. This study compared and analyzed the maximum tumor diameter measured by ultrasound after NAC6 with that measured by pathology after surgical resection. The maximum tumor diameter measured by ultrasound was 1.20 (0.70, 2.10) cm, while the maximum diameter measured by pathology was 1.20 (0.10,2.15) cm, demonstrating a significant positive correlation between the two (*r*=*0.626*, *P*<0.05). Therefore,ultrasound can effectively monitor tumor size and its changes before and after NAC.

Studies (25) have shown that the clinical T stage of the tumor is the most important predictor of whether breast cancer patients can achieve pCR after NAC. In this study, the maximum tumor diameter measured by ultrasound before NAC was used to perform clinical T staging. The results revealed that the pCR rates of the T1 stage(≤2cm), T2 stage(> 2cm, ≤5cm), and T3 stage(> 5cm) were 51.7%, 24.6%, and 3.7%, respectively. These findings also confirmed that a higher clinical T stage indicated a lower rate of achieving pCR. Unfortunately, among the 249 patients in this study, only a minority (56,22.5%) were in clinical stage T1, while more patients were in stage T2 (166,66.7%), and some patients were in stage T3 (27,10.8%). This is similar to the results of a previous study (26) and indirectly emphasizes the importance of early detection and early diagnosis of breast cancer.

The change in tumor burden is a key point of clinical evaluation during cancer treatment, and tumor shrinkage or progression are important evaluation indexes. The changes in maximum tumor diameter after NAC2, NAC4, and NAC6 were quantitatively evaluated according to RECIST. The results showed that the RECIST assessment was valid (CR+PR) in 108 cases (43.3%) after NAC2, 174 cases (69.0%) after NAC4, and 194 cases (77.9%) after NAC6. Multivariate logistic regression analysis of the training set showed that the RECIST assessment after NAC2, NAC4, and NAC6 were all independent predictors of pCR. Although previous literature (28–30) has reported concentric shrinkage patterns or non-concentric shrinkage patterns such as nodules and nests after NAC, RECIST assessment remains significant in clinical practice due to its effectiveness, simplicity, low cost, and easy interpretation of results.

In this study, among the 71 (28.5%) pCR patients, 38 showed no residual malignant cells in histopathology, and 34 showed only intraductal carcinoma. However, after NAC6, only 30 patients showed no residual lesions by ultrasonography. This discrepancy may be attributed to tumor cells being broken by hypoxia, leaving only fibrotic and collagen tissue, which may be misinterpreted by routine ultrasound.

Posterior shadow is usually thought to be due to the increased absorption of sound waves caused by the tumor containing more collagen fiber components and larger tissue hardness. Therefore, this feature is usually used as a malignant sign to describe the ultrasound characteristics of the tumor (31, 32), indicating a greater risk of malignancy. The results of this study showed that patients with posterior shadows were less likely to achieve pCR. The results of this study are consistent with the malignant biological behavior of tumors.

In recent years, biological factors surrounding the treatment and prognosis of breast cancer have emerged, with the most extensively studied factors being the expression of ER, PR, HER2, and Ki67. ER and PR are hormone-dependent tumor cells whose functions are regulated by the endocrine system. Colleoni et al. (33) reported that patients with hormone receptor-negative were 12 times more likely to achieve pCR than hormone receptor-positive patients. Our results also revealed that people with negative PR are more likely to achieve pCR. However, hormone receptor-negative patients are not sensitive to endocrine therapy and have a poor prognosis (34, 35). HER2 is a proto-oncogene that is normally inactive but can be activated by certain factors in and out of the body. HER2 overexpression is directly related to tumor growth, invasion, and prognosis (36). According to the literature (37), HER2 positivity is an indicator of pCR, but the same results were not observed in our study after multivariate logistic regression analysis. This may be due to the limited sample size and confounding effects between factors. Ki67 is a large molecular nuclear protein associated with cell proliferation. High proliferation indicates active proliferation and high aggressiveness of tumor cells. Liu, Q et al. (38) pointed out that patients with higher Ki67 were more likely to achieve pCR, while our results showed no significant correlation between Ki67 and pCR. This discrepancy may be attributed to different selected critical values for Ki67. Therefore, the ideal critical value of Ki67 requires further study.

Our study is a retrospective single-center clinical study, there may be potential selection bias.Due to individual differences between patients, changes in ultrasound equipment and examination parameters may affect the quality and consistency of images. The ultrasound features involved were grayscale and color Doppler characteristics. In future studies, the sample size should be expanded and the accuracy of multimodal ultrasound in predicting the efficacy of NAC should be explored by combining elastography, contrast-enhanced ultrasound, and radiomics. In addition, the prediction model constructed in this study was only validated internally and lacks external validation. Therefore, the validity of this model needs to be further evaluated.

## 5 Conclusion

Patients with breast cancer treated with NAC were more likely to achieve pCR when ultrasound showed a small tumor, the posterior echo indicated no shadow, the RECIST was valid, and pathology showed a negative PR. The prediction model of pCR after different cycles of NAC established by the combination of ultrasound and clinicopathology demonstrated high clinical differentiation and calibration, offering a significant clinical application value. The online dynamic nomogram model also provides a more convenient tool for clinicians. The possibility of pCR can be predicted early after NAC2.

## Data Availability

The original contributions presented in the study are included in the article/supplementary material. Further inquiries can be directed to the corresponding author.
